# Utility of esophageal gastroduodenoscopy at the time of percutaneous endoscopic gastrostomy in trauma patients

**DOI:** 10.1186/1749-7922-2-18

**Published:** 2007-07-05

**Authors:** James M Haan, Grant V Bochicchio, Thomas M Scalea

**Affiliations:** 1 Department of Surgical Critical Care, R Adams Cowley Shock Trauma Center, University of Maryland School of Medicine, Baltimore, Maryland, USA

## Abstract

**Background:**

The utility of esophagogastroduodenoscopy (EGD) performed at the time of percutaneous endoscopic gastrostomy (PEG) is unclear. We examined whether EGD at time of PEG yielded clinically useful information important in patient care. We also reviewed the outcome and complication rates of EGD-PEG performed by trauma surgeons.

**Methods:**

Retrospective review of all trauma patients undergoing EGD with PEG at a level I trauma center from 1/01–6/03.

**Results:**

210 patients underwent combined EGD with PEG by the trauma team. A total of 37% of patients had unsuspected upper gastrointestinal lesions seen on EGD. Of these, 35% had traumatic brain injury, 10% suffered multisystem injury, and 47% had spinal cord injury. These included 15 esophageal, 61 gastric, and six duodenal lesions, mucosal or hemorrhagic findings on EGD. This finding led to a change in therapy in 90% of patients; either resumption/continuation of H_2 _-blockers or conversion to proton-pump inhibitors. One patient suffered an upper gastrointestinal bleed while on H2-blocker. It was treated endoscopically. Complication rates were low. There were no iatrogenic visceral perforations seen. Three PEGs were inadvertently removed by the patient (1.5%); one was replaced with a Foley, one replaced endoscopically, and one patient underwent gastric repair and open jejunostomy tube. One PEG leak was repaired during exploration for unrelated hemorrhage. Six patients had significant site infections (3%); four treated with local drainage and antibiotics, one requiring operative debridement and later closure, and one with antibiotics alone.

**Conclusion:**

EGD at the time of PEG **may add **clinically useful data in the management of trauma patients. Only one patient treated with acid suppression therapy for EGD diagnosed lesions suffered delayed gastrointestinal bleeding. Trauma surgeons can perform EGD and PEG with acceptable outcomes and complication rates.

## Background

Enteral nutrition has been shown to be far superior to parenteral nutrition following injury. Percutaneous endoscopic gastrostomy (PEG) is a common procedure for enteral access following severe injury [1-4]. PEG is a relatively straightforward procedure that can be performed at the bedside and has become the procedure of choice in many trauma centers [5-11]. Performing an esophago-gastroduodenoscopy (EGD) at the time of PEG placement may be helpful, but the exact role is not clear. Some series exist, but a large percentage of those patients underwent PEG for an indication other than trauma [4-10,12,13]. The yield of EGD at the time of PEG in those series was variable. Trauma patients may be at special risk for incidental gastrointestinal pathology as the high stress state following injuries such as traumatic brain injury or spinal cord injury may predispose patients for conditions such as stress gastritis or duodenal ulcers despite prophylaxis.

In 2001, the trauma service at the R Adams Cowley Shock Trauma Center began performing its own PEGs. EGD was performed at the time of all PEG placements. We retrospectively reviewed our experience over two years in order to determine the clinical utility of EGD at the time of PEG placement. In addition, we wished to determine whether general surgeons on the trauma service could safely and effectively perform both EGD and PEG placement.

## Methods

In January 2001, the attending surgeons on the trauma service at the R Adams Cowley Shock Trauma Center began performing EGDs and PEGs. Previously to this, PEGs had been placed by the surgical endoscopy service in the Department of Surgery at the University of Maryland School of Medicine. Six surgeons from the trauma service were credentialed to place PEGs. These surgeons had significant experience during residency and/or fellowship training sufficient to credential them for EGD with PEG placement. A PEG service was created and referrals made to it.

By protocol, patients had to tolerate nasogastric feedings before PEG placement was considered. A full survey of the esophagus, stomach and proximal duodenum was attempted before PEGs were placed. PEG tubes were inserted using the pull method modified from the original technique as described by Ponsky using a commercially available kit [7].

We retrospectively reviewed the 2-1/2 year period, January 2001 through June 2003. Charts were reviewed and the basic trauma demographic data obtained such as age, sex, mechanism of injury and indication for PEG placement. Findings on EGD were reviewed as well as treatment recommendations based on the endoscopic findings. Data was then analyzed. The University of Maryland School of Medicine Institutional Review Board approved the study.

## Results

During the study period, 210 patients underwent EGD with PEG placement at the R Adams Cowley Shock Trauma Center. All patients were admitted following injury. Seventy percent had traumatic brain injury and failed a swallowing evaluation by a Speech Language Pathologist. Ten percent of patients had multisystem injury with dysphasia, and 23% had spinal cord injury with dysphagia or were unable to tolerate sufficient PO nutrition to maintain adequate caloric intake. Patient characteristics are depicted in Table 1.

**Table 1:** Patient Characteristics

Total patients	210
Male	77%
Mean age (years)	37
Age range (years)	14–86

PEG placement was successful in 98% of patients. Full endoscopic survey was successfully performed in 99% of patients. PEG placement was aborted in three patients due to an inadequate red light reflex. Full endoscopic survey was unsuccessful in four patients. One patient was unable to tolerate prolonged insufflation and in three patients, the endoscopist was unable to intubate the duodenum.

Unsuspected endoscopic findings occurred in 37% of the patients undergoing full EGDs. These included 15 esophageal, 61 gastric, and 6 duodenal lesions. The specific findings are detailed in Table 2. Unsuspected endoscopic findings occurred in 35% of patients with traumatic brain injuries, 47% of patients with spinal cord injuries, and 10% of patients with multisystem injury and dysphagia (Table 3).

**Table 2:** Pathologic Findings on EGD

Esophagus	*n (%)*	Stomach	*n (%)*	Duodenum	*n (%)*
Esophagitis	15 **(7%)**	Gastritis	24 **(11%)**	Duodenal ulcer	6 **(3%)**
		Bleeding Ulcer	5 **(2%)**		
		Recent ulcer	24 **(11%)**		
		Hiatal hernia	5 **(2%)**		
		Mass lesion	3 **(1%)**		

N = with findings on EGD

% = % of total population

**Table 3:** Positive Finding on EGD Based on Diagnosis

	Spinal Cord	Brain Injury	Multisystem
Positive EGD	47%	35%	33%
% of Population	23%	70%	10%

The majority of endoscopic findings were mucosal lesions in the stomach or unsuspected ulcers. This lead to a change of therapy in 90% of patients with positive endoscopic findings. These patients were all tolerating tube feedings. By protocol at our institution, gastric prophylaxis is discontinued when gastric feedings are tolerated. Patients found to have healing ulcers were continued on H_2 _blockade for an additional month. However, 50% of patients had significant gastritis, ulceration or evidence of acute pathology and were converted to proton pump inhibitor therapy. One of these patients developed an upper GI bleed despite conversion to proton pump inhibitors. This patient was successfully treated endoscopically. Three polypoid lesions were seen. Due to the patient's age and severity of brain injury, these were not treated at the time, but patients were referred for repeat endoscopy with evaluation and biopsy when they had recovered.

Complication rates were low. There were no iatrogenic visceral perforations in these patients. Three PEGs were inadvertently removed by the patient (1.5%). One was replaced by a Foley catheter, one was replaced endoscopically, and one patient underwent laparotomy, gastric repair and open jejunostomy tube placement. One PEG leak was repaired during exploration for unrelated hemorrhage. Six patients (3%) had PEG site infections. One was successfully treated with IV antibiotics alone, four were treated with local incision and drainage and antibiotics. One patient however, required operative debridement of the abdominal wall with delayed primary closure.

## Discussion

The technique for PEG placement has evolved since is original description and there is a large volume of literature describing the evolution of the technique [1-13]. PEGs have become the procedure of choice for enteral access in patients requiring long-term tube feedings after trauma [5-11]. PEGs are a relatively straightforward procedure that can often be performed at the bedside, in the Intensive Care Unit or Intermediate Care Unit. Feedings can be started soon after PEG placement, thus minimizing the risk of malnutrition. PEGs are relatively safe with an accepted complication rate of <6–30%. [8,11].

While many series exist concerning the use of PEG placement, most of these series contain a paucity of trauma patients. Trauma patients may be different for a number of reasons. Trauma creates a catabolic state with marked increase in nutritional needs. Infection is a common occurrence in the Intensive Care Unit following injury. Patients with major torso trauma may be unable to tolerate enteral nutrition early on, condemning them to TPN or even worse, no nutritional support at all. The high stress ulceration rate following injury could predispose trauma patients to a significant incidence of incidental death or upper gastrointestinal pathology despite routine use of gastrointestinal prophylaxis.

Unsuspected gastrointestinal pathology can be seen at the time of PEG placement. Several studies have described unsuspected pathologic findings in 10 to 60% of patients. Scott et al reported medical treatment changes based on EGD findings in approximately 38% of patients. In 5% of these patients, pathologic ulceration and pyloric stenosis was found, requiring duodenal feeding due to gastric outlet obstruction. A prior study from this institution described unsuspected pathologic findings in 38% of surveyed patients. This occurred in the esophagus 7% of the time, stomach 24% and duodenum 7%. In that series however, the patients studied included trauma patients and those with head and neck cancer and/or stroke. In our series of only trauma patients, we found only an eight percent incidence of duodenal pathology and no unsuspected gastric outlet obstructions. As we require that patients scheduled for PEG tolerate gastric feedings, no patients with unsuspected obstruction were scheduled for PEG placement.

We retrospectively reviewed 210 patients who underwent attempted PEG placement with survey endoscopy over a 2-1/2 year period. Our rate of unsuspected gastrointestinal pathology was 37%, not different from many other reports. The majority of these patients had non-specific mucosal changes and/or inflammatory injury consistent with stress pathology. It is perhaps not surprising that stress gastritis and/or ulceration would be the most frequent finding as over two-thirds of our patients sustained spinal cord injury or traumatic brain injury, presumably placing them at high risk for bleeding. All patients at our institution undergo routine GI prophylaxis at the time of admission. While the rate of unsuspected pathology is not substantially different than the literature, it is not clear from those studies, what percent of those patients underwent routine prophylaxis.

It is perhaps reassuring to note that although there was a substantial incidence of unsuspected GI pathology, the majority were controlled or healing with standard stress prophylaxis. In those patients, the prophylaxis was simply continued. Approximately half of the patients had acute changes at the time of EGD. These patients were changed to proton pump inhibitor therapy. Only one failed medical management with an upper GI bleed, which was successfully treated endoscopically.

It would seem then that there is a real role for surveillance endoscopy at the time of PEG placement. These unsuspected lesions are clinically significant and in 90% of patients, therapy was altered in some manner.

The overall complication rate in our series was 4.5%. There were no iatrogenic complications at the time of PEG placement. Only two patients required open therapy. One patient who inadvertently pulled their PEG out required laparotomy, gastric repair and jejunal feeding tube replacement. A second patient had an unsuspected PEG leak repaired at the time of laparotomy for unrelated hemorrhage. Other complications were relatively minor. We were concerned about the fact that three PEGS were pulled out by the patient and have modified our protocol since. We now currently routinely place abdominal binders over the PEG site and have the tube exit laterally out of the binder. If a patient with brain injury or multi-system trauma gets agitated and grabs the feeding tube, they merely pull the adapter out of the PEG rather than removing the PEG. Other centers have described the use of T-fasteners for agitated patients to maintain the PEG tract if inadvertently removed. Unfortunately many of these products have been recalled and are not currently available.

General surgeons specializing in trauma and critical care seem to be able to safely place tubes. In our series, PEG placement was successful in 98% of patients and full endoscopic survey was successful 99% of the time. This is not different than the 97% successful PEG placement and 99% successful endoscopic survey reported from our own institution when PEGs were performed by the surgical endoscopy service. EGD and PEG placement are basic skills and should be able to be mastered by any well-trained surgeon. Adding full surveillance endoscopy to the examination at the time of PEG placement should only add a few additional minutes to the procedure and requires basic endoscopic maneuvers most of the time. This does not require advanced endoscopic skills or intervention, which almost certainly should be performed by a specially trained endoscopist.

## Conclusion

EGD at the time of PEG adds clinically useful information in a substantial portion of trauma patients, potentially decreasing risks of GI bleeding. Both EGD and PEG placement can successfully be performed in the vast majority of trauma patients by general surgeons on the trauma service.

## Authors' contributions

JMH drafted the manuscript and was responsible for the majority of data acquisition. GVB assisted with data acquisition. TMS provided editorial support. All authors read and approved the final manuscript.
